# Loss to Follow-Up Risk among HIV Patients on ART in Zimbabwe, 2009–2016: Hierarchical Bayesian Spatio-Temporal Modeling

**DOI:** 10.3390/ijerph191711013

**Published:** 2022-09-02

**Authors:** Zvifadzo Matsena Zingoni, Tobias Chirwa, Jim Todd, Eustasius Musenge

**Affiliations:** 1 Division of Epidemiology and Biostatistics, School of Public Health, Faculty of Health Sciences, University of the Witwatersrand, Johannesburg 1619, South Africa; 2 Department of Population Health, London School of Hygiene and Tropical Medicine, London WC1E 7HT, UK

**Keywords:** HIV prevention, LTFU risk, ART, conditional autoregressive models, spatio-temporal, poisson regression

## Abstract

Loss to follow-up (LTFU) is a risk factor for poor outcomes in HIV patients. The spatio-temporal risk of LTFU is useful to identify hotspots and guide policy. Secondary data on adult HIV patients attending a clinic in provinces of Zimbabwe between 2009 and 2016 were used to estimate the LTFU risk in each of the 10 provinces. A hierarchical Bayesian spatio-temporal Poisson regression model was fitted using the Integrated Nested Laplace Approximation (INLA) package with LTFU as counts adjusting for age, gender, WHO clinical stage, tuberculosis coinfection and duration on ART. The structured random effects were modelled using the conditional autoregression technique and the temporal random effects were modelled using first-order random walk Gaussian priors. The overall rate of LTFU was 22.7% (95%CI: 22.6/22.8) with Harare (50.28%) and Bulawayo (31.11%) having the highest rates. A one-year increase in the average number of years on ART reduced the risk of LTFU by 35% (relative risk (RR) = 0.651; 95%CI: 0.592–0.712). In general, the provinces with the highest exceedance LTFU risk were Matabeleland South and Matabeleland North. LTFU is one of the drawbacks of HIV prevention. Interventions targeting high-risk regions in the southern and northern regions of Zimbabwe are a priority. Community-based interventions and programmes which mitigate LTFU risk remain essential in the global HIV prevention campaign.

## 1. Introduction

The human immunodeficiency virus (HIV) is a complex retrovirus that progressively destroys the body’s immune system, making it harder for infected individuals to battle other infections [1]. However, there have been several HIV prevention programmes and policies to fight the pandemic [2]. Antiretroviral therapy (ART) has been the backbone of HIV prevention and has left an indelible mark in the HIV fight, but requires consistent, regular maintenance of the regime. ART aims to suppress the viral load, thus decreasing the aggression of the immune system and enabling it to recover [3]. Slowing down the HIV progression and suppressing the virus to undetectable levels using the ART treatment results in un-transmittable HIV; hence, preventing HIV transmission in the general population [4].

Most countries have achieved the UNAIDS 90–90–90 fast-track targets and are now setting their focus on the 95–95–95 targets for HIV diagnoses, HIV treatment and viral suppression [5]. The last target is the prime focus of HIV prevention and control; however, achieving it is strongly dependent on how well HIV patients are adhering to ART. One of the challenges faced in HIV prevention programmes, in particular the ART programme, is the loss to follow-up (LTFU), which is one of the “leakages” of patients from the ART programme [6]. As patients become LTFU, their ART adherence is compromised.

In Zimbabwe, the overall adult HIV prevalence at the country level was estimated at 13.7%; however, the HIV prevalence varies by province due to significant heterogeneity in social, cultural and population dynamics which are mainly influenced by the geographical correlation of the epidemic. Like other sub-Saharan African countries, Zimbabwe has faced a significant rate of attrition mainly due to LTFU in its ART prevention programme [7,8]. Some of the underlying reasons assumed to motivate LTFU had been misconceptions over ART benefits, health literacy, cultural beliefs and inclination toward traditional medicine [9]. Zimbabwe implemented the decentralization of the ART services model from high levels of care to primary health care facilities (PHC) in 2012/13 and at the end of 2017, there were approximately 1566 health facilities offering ART [10]. The decentralization of these health facilities resulted in increased access to ART for all those in need and improved ART uptake in the country, but there were challenges too.

Several studies have looked at attrition in the Zimbabwe National ART programme [7,8] but none described the spatio-temporal patterns of LTFU before and after ART service decentralization at the national level using routinely collected programme data. This study hypothesised that there might have been changes in the LTFU patterns after ART decentralization which may vary across different regions in Zimbabwe over time. Describing the geographical distribution of LTFU risk through mapping is quite useful for policymakers and programme managers to have a pictorial view of the high-risk regions and to be able to guide resource distribution and implement region-specific interventions or policy formulation [11]. Besides, the gained knowledge helps in the formulation of hypotheses for further research and guides surveillance in high-risk regions that improve ART patients’ retention. Therefore, this study set out to describe the spatio-temporal risk of LTFU at the province level from 2009–2016 and pinpoint cold and hot-spot regions of LTFU in Zimbabwe using the hierarchical Bayesian spatio-temporal Poisson regression model.

## 2. Materials and Methods

### 2.1. Study Design

This study was a secondary data analysis of routinely collected HIV monitoring data compiled through the electronic patient management system (ePMS) which is a representative sample of all people living with HIV (PLWHIV) in Zimbabwe [10].

### 2.2. Study Site

This study was done in Zimbabwe. The country is made up of eight provinces (Matabeleland North, Matabeleland South, Masvingo, Midlands, Mashonaland East, Mashonaland Central, Mashonaland West and Manicaland) and two metropolitan cities (Harare and Bulawayo).

### 2.3. Data Source

The data came from the ePMS in Zimbabwe from 2009 to 2016 and only information for adults aged 15 years and above was used. The database captures HIV patients’ monitoring information and outcomes. LTFU was defined as having 90 days since the last appointment and was analysed as count data. The other covariates were mean age at ART initiation, the mean duration of patients on ART, the proportion of females, the proportion who were in WHO stage III/IV and the proportion of patients who had confirmed tuberculosis. The province was the spatial unit of analysis during the year in which ART initiation provided the temporal patterns.

### 2.4. Statistical Analysis

#### 2.4.1. The Bayesian Spatio-Temporal Poisson Regression Model Specification

To estimate the LTFU risk after ART initiation and quantify the risk associated with LTFU and age, duration whilst on ART, being a female, having tuberculosis and being in WHO stage III/IV, a hierarchical Bayesian spatio-temporal Poisson regression model was fitted to take into account the spatial dependence among neighbouring regions. A Bayesian spatio-temporal model consists of three components: the likelihood function (i.e., the data distribution given the parameters), the linear predictor function (i.e., the relative risk structure based on fixed and random effects including the underlying spatial trend), and the prior distribution functions (i.e., the prior distribution of the unknown parameters in the model).

##### Likelihood Function

Let the LTFU counts follow a Poisson distribution with the following probability density function:

(1) $$\begin{array}{l} P\left(Y_{it}\right)=\frac{{\left(\lambda_{it}E_{it}\right)}^{Y_{it}}}{Y_{it}!}\exp\left(-\lambda_{it}E_{it}\right)\;;\;i=1,2,\ldots ,n\;\mathrm{and}\;t=1,2,\ldots ,Z\\ ∴Y_{it}\sim \mathrm{Poisson}\left(\lambda_{it}E_{it}\right) \end{array}$$

where $Y_{it}$ defines the observed LTFU counts at region i at the time t. The model assumes that the mean of the observed LTFU counts $\left(\mu_{it}\right)$ is a product of the expected count $\left(E_{it}\right)$ and the relative risk $\left(\lambda_{it}\right)$, i.e., $\mu_{it}=\lambda_{it}E_{it}$.

##### Linear Predictor Function

The linear predictor model describes the underlying structure of the relative risk in relation to the fixed and random effects. The linear predictor model included the spatio-temporal random effects as defined by the convolutional conditional autoregressive (CAR) model proposed by Besag-York-Mollie (BYM) [12] with separable structured and unstructured spatial random effects. All known parameters were assumed some prior information. The model is specified as follows:

(2) $$\log\left(\lambda_{it}\right)=\log\left(E_{it}\right)+\alpha +X_{i}^{T}\beta +u_{i}+v_{i}+\gamma_{t}+\psi_{it}$$

The model parameters and prior are defined in Table 1 below:

##### The Space-Time Interaction Terms

The space-time interactions used are described in more detail elsewhere [14]. The type I interaction model was a product of the temporal and unstructured spatial random effects. This interaction term had a structure matrix of $R_{\psi }=R_{\gamma }\times R_{v}$. This interaction term was assumed to follow an independent Gaussian distribution, $\psi_{it}\overset{iid}{\sim }N\left(0,\sigma_{\psi }^{2}\right)$. The type II interaction was a combination of unstructured spatial effects and temporal effects. The interaction structure matrix was defined as $R_{\psi }=R_{\gamma (rw1)}\circ R_{v(iid)}$. The neighbouring unstructured spatial component was assumed to be independent and the temporal effects followed a first-order random walk. The interaction term assumed that the structured spatial units were time-correlated but with independent time trends in different areas. The type III interaction term combines the structured component and the temporal effects. The interaction structure matrix was defined as $R_{\psi }=R_{\gamma (iid)}\circ R_{u(CAR)}$. The spatial neighbouring of the interaction terms was defined through the CAR model specification. The model assumed that $t^{\prime }\neq t\in \gamma_{t}$ are independent across the structured spatial structures.

The full posterior model specifications and derivations implemented in this study and the R code are provided as Supplement Materials, Sections S1 and S2.

#### 2.4.2. Bayesian Model Fitting and Model Comparison

Hierarchical Bayesian spatial-temporal Poisson regression models were fitted in R using the Integrated Nested Laplace Approximation (INLA) which is a computationally less intensive method that approximates Bayesian inference. The INLA method uses analytical approximation and numerical integration to obtain the posterior distribution of the parameters. To compare the spatio-temporal models, the Deviance Information Criterion (DIC) values were used. The model with the lowest DIC value was selected as the best fitting model with the lowest complexity and was the one used for the interpretation of the results [15].

## 3. Results

### 3.1. Loss to Follow-Up Trend and Descriptive Statistics

**Figure 1:**
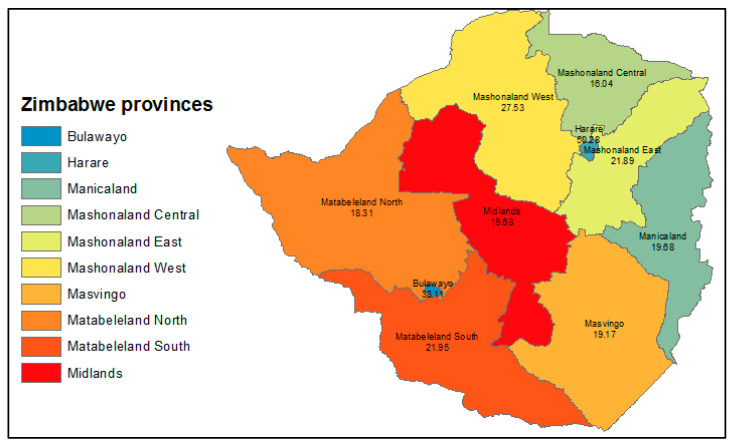
The choropleth distribution of loss to follow-up stratified by province, 2009–2016.

The overall rate of LTFU between 2009–2016 was estimated at 22.7% (95% CI: 22.6–22.8). The highest LTFU rate was observed in Harare province (50.28%) and Bulawayo (38.11%), Figure 1.

The baseline characteristics of the study participants are summarised in Table 2. In each province, there were more females (>50%) than males. The average age was approximately 37 years and those patients who had TB coinfections were below 4% in each province. There were more than 50% of the patients in WHO clinical stage III/IV except for Bulawayo, Masvingo, Midlands and Matabeleland provinces which had less than 50%. The median time on ART ranged from 2.6–3.6 years across the provinces.

**Figure 2:**
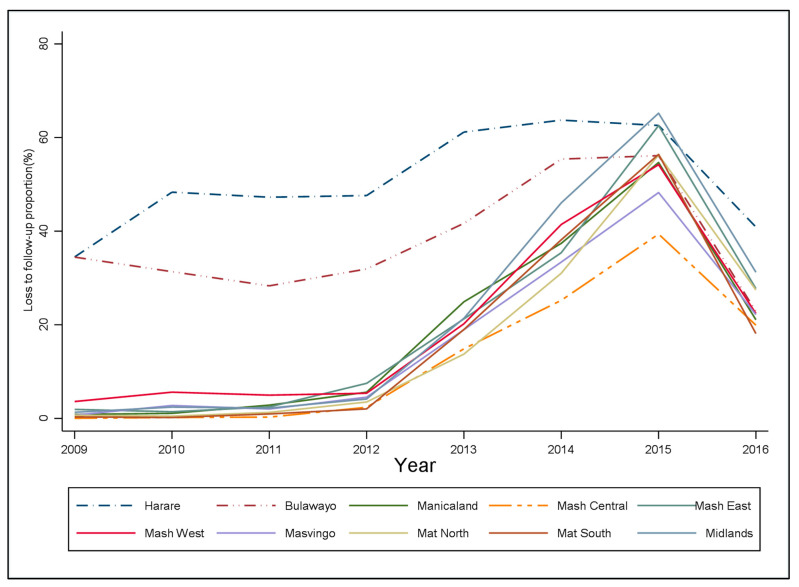
The trend analysis of loss to follow-up stratified by province.

The trend analysis of LTFU stratified by the province is displayed in Figure 2. In general, LTFU cases were low between 2009–2012 followed by an increase from 2012–2015 then a drop in 2016. LTFU cases were highest in Harare province over time and Mashonaland Central province had the least LTFU cases over time. The observed and predicted LTFU rates are shown in the Supplementary material, Figures S1 and S2.

### 3.2. Factors Associated with LTFU

Table 3 summarises the results for the hierarchical Bayesian spatio-temporal Poisson regression models at the province level. Adding the temporal random effects significantly improved the model (DIC = 3079.93 vs. DIC = 9367.17). The interaction term further improved the model significantly as the DIC values became much lower (type I interaction DIC = 787.33; type II interaction DIC = 784.08; type III interaction DIC = 79.03). The type II interaction model was the best fitting model.

Regarding the risk factors of LTFU in this study, the type II interaction model did not detect any significant association for the aggregated gender variable, the proportion who had tuberculosis and the proportion who were in WHO stage III/IV. However, the average duration whilst on ART treatment had a protective effect on LTFU. A one-year increase in the average number of years on ART reduced the risk of LTFU by 35% (relative risk (RR) = 0.651; 95%CI: 0.592–0.712).

### 3.3. Spatio-Temporal Patterns of LTFU

**Figure 3:**
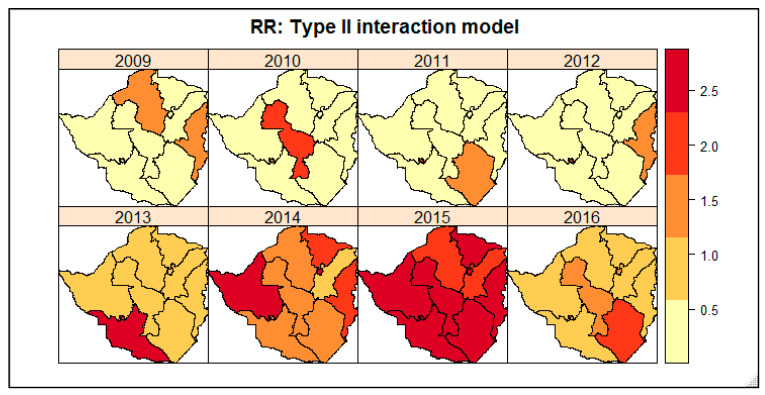
Spatio-temporal distribution trellis plots of the LTFU relative risk at the province level.

Figure 3 displays the spatio-temporal distribution of the LTFU RR estimated from the type II interaction model from 2009–2016 at the province level. The darker colour shows the provinces with the highest RR values and the lighter colour shows otherwise.

The spatio-temporal distribution of the LTFU RR varied across provinces over time. Between 2009 and 2012 the risk of LTFU was very low and was concentrated in a few provinces. The provinces which showed a high risk of LTFU were Mashonaland West and Manicaland in 2009; Midlands in 2010; Masvingo in 2011 and Manicaland in 2012. From 2013 to 2016, the LFTU risk was relatively high in all provinces. In 2013, the province with a high risk of LTFU was Matabeleland South and in 2014 the province with a high risk of LTFU was Matabeleland North. Due to the increase in LTFU cases in 2015, all provinces had an LTFU RR of at least 2 times. In 2016 the LTFU cases decreased and the province with a high risk of LTFU was Masvingo.

### 3.4. Provinces with Exceedance LTFU Risk

**Figure 4:**
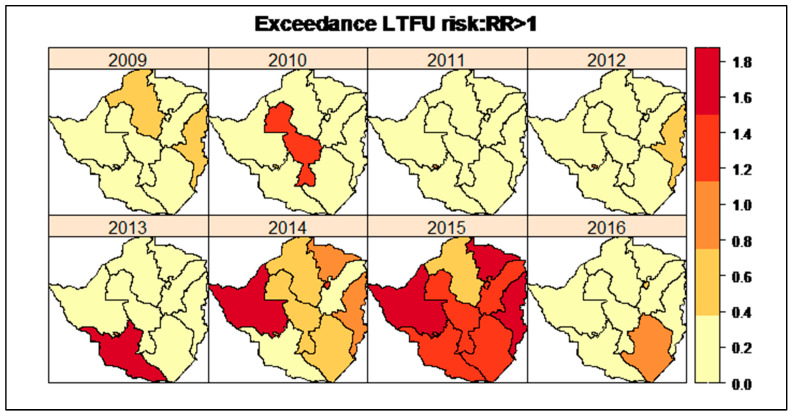
The LTFU exceedance risk trellis plots overtime per province.

Often, it is of interest to estimate the excess RR of an outcome in a region based on a threshold value T. The “exceedance risk” helps identify the regions with a high risk of LTFU. In this study, a threshold value of T=1 was considered. Figure 4 shows regions that had an exceedance risk of LTFU RR>1 over time. The darker colour shows the provinces with the highest exceedance risk values and the lighter colour shows otherwise.

The provinces with the highest exceedance LTFU risk were Matabeleland South in 2013 and Matabeleland North in 2014. In 2015, most provinces had high exceedance risk; however, the most outstanding provinces were Matabeleland North, Mashonaland Central and Manicaland. In 2016, Masvingo province had the highest exceedance LTFU risk.

## 4. Discussion

This study aimed to describe the spatio-temporal risk of LTFU at the province level from 2009 to 2016 and pinpoint cold- and hot-spot regions of LTFU in Zimbabwe among adult HIV patients to guide policy. A hierarchical Bayesian spatio-temporal Poisson regression model was used assuming CAR priors for the structured spatial random effects and Gaussian first-order random walk prior to the temporal random effects. The model adjusted for the space-time interaction terms as random effects and also fixed effects covariates.

In general, the trend plot of the LTFU over time showed a general constant increase in LTFU cases from 2009 to 2012. The low and constant LTFU rate in 2009–2012 can be explained by the timing of the ePMS implementation in Zimbabwe. The ePMS was implemented in 2012 and all the data for previous years were captured retrospectively although those after 2012 were captured prospectively [6]. This means there might have been selection bias in capturing records of patients before 2012 due to high patient record volumes and most of those who might have been LTFU were left out in the electronic database. Moreover, since the 2009–2012 records were captured retrospectively, there is a possibility of missing records or toned records which made the data retrieval very difficult [6,16].

This study observed a swift increase in the LTFU rate from 2013 to 2015 which is a period after the introduction of a differential monitoring mechanism. This increase in the LTFU rate could have been an improvement in reporting as information was now reported prospectively [16]. Contrarily, the decentralization of ART services from higher levels of care to lower levels of care might have been the reason for the upsurge of LTFU cases. Results show that the highest LTFU rates were experienced in Harare and Bulawayo (Figure 2) which have mostly high levels of care facilities. This means many patients might have self-transferred from these higher levels of care in metropolitan provinces to PHC facilities without being tracked or linked in the ePMS [6,17]. These “silent transfers” may have been administratively misclassified as LTFU when in fact the patients are already re-engaged in care at a different health facility [17]. Another explanation for the increase in LTFU between 2013–2015 could be that as patients were enrolling in nearby health facilities, these primary health care facilities might have been poorly prepared to retain patients on care [8].

Moreover, during the 2013–2015 period, the HIV management guidelines changed. The CD4 cell counts cut-off for ART initiation was increased from 350 cells/μL in 2012 to 500 cells/μL in 2013 [2,8]. This resulted in a high influx of HIV patients in the HIV prevention programme. Studies have shown that patients who start ART with a higher CD4 cell count are generally well which can trigger non-adherence or low-risk perception to long-term therapy; hence, become LTFU [8,18,19]. The country also introduced HIV strategies like “test and treat all” which targeted the high-risk groups such as those with tuberculosis co-infection and serodiscordant partners, and pregnant women. These sub-population groups were known to have a high risk of LTFU as some could demise if they become critically ill or default from treatment due to denial, especially serodiscordant partners [8].

The aggregated information of females, having tuberculosis and being in WHO stage III/IV were not associated with the risk of LTFU in this study based on the type II interaction model. This contrasts with findings from previous studies [6,7,20,21,22,23]; however, these studies were done at individual-level analysis and not aggregated as this current study. The use of aggregated data might have obscured the detection of a significant association due to the reduced sample size. Moreover, in this study WHO clinical stage was combined into I/II and III/IV while other studies analysed each WHO clinical stage individually or by combining WHO stages 1, II, and III and comparing it to WHO stage IV [8]. Secondly, most studies that have reported an increased risk of the WHO clinical stage in predicting LTFU or attrition have used time-to-event models which are different to those we conducted in this current study which used a Poisson regression [6,7,8]. Lastly, the predicted outcome varies with other studies, some studies predict attrition (mortality, LTFU, drop-out and withdrawal) [7,8] but this study looked at only LTFU. However, it was interesting to detect that the longer the patient was initiated on ART, the lesser the risk of becoming LTFU. Interventions that strengthened reduced LTFU among new patients, such as strengthening electronic databases which show status at all health facilities, integrating services, community-based interventions, and making follow-ups with patients who meet the LTFU criteria, should be prioritized [24].

The spatio-temporal pattern of LTFU risk varied across provinces and the risk of LTFU was high in 2015 for the whole country. The most persisting regions with high exceedance LTFU risk were Matabeleland North and Matabeleland South. This finding could be explained by the highest HIV prevalence [25] associated with these regions. From the Zimbabwe Population-based HIV impact assessment (ZIMPHIA) survey in 2015/16, Matabeleland South and Matabeleland North provinces had a high HIV prevalence of 22.3% and 20.1%, respectively [25]. The higher the HIV prevalence the higher the risk of becoming LTFU; hence, a dose-response relationship existed between HIV infection and the risk of LTFU exists in these regions. Since these regions were known to have an increased number of HIV/tuberculosis coinfections [26], the patients on ART in these regions were more likely to have poor viral suppression [27]. This may increase the risk of treatment defaulting which subsequently becomes LTFU and some may eventually die. Such regions require targeted area-specific interventions which minimise LTFU and retain patients on care [9].

The strengths of this study included the use of hierarchical Bayesian spatio-temporal Poisson regression to determine the risk of LTFU at the province level [12]. This study was one of the few studies which have used spatio-temporal modeling in HIV prevention to highlight epidemiological spatio-temporal patterns of LTFU risk with application to the Zimbabwe HIV population. The modelling approach implemented in this study utilized probability to measure uncertainty in estimates inferences; hence, it was robust. Nonetheless, there were aerial data structure issues associated with aggregated spatial data. The data used in this study were created at a higher administrative unit or province, and the results reported in this study may be different if a lower administrative unit such as districts had been used. However, the analysis at the provincial level allowed the formulation of new hypotheses for future studies. Another limitation of this study was the use of the ePMS database which was a sample of the total population of HIV-positive patients on ART in Zimbabwe and this might have resulted in selection and negatively affected the generalizability of our findings. However, the data used had been reported to be a representative sample of the HIV population in Zimbabwe [16].

## 5. Conclusions

LTFU remains a challenge in HIV monitoring and prevention. The regions more at risk were the southern and northern regions of Zimbabwe and these findings were comparable to the nation’s HIV prevalence and HIV/tuberculosis coinfection geographical distribution. Our analysis puts forward that although tremendous progress might have been made regarding HIV prevention, there was more work that needed to be done to retain patients on care. LTFU is one of the drawbacks of HIV prevention which impedes the achievement of certain targets such as the UNAIDS. There is a need to develop an intervention, especially in high-risk regions, to minimise the risk of LTFU, particularly in the southern and northern regions of Zimbabwe. Implementation of area-specific targeted interventions in resources-constrained countries to sustain the current HIV interventions is crucial. Once the LTFU risk has been mitigated, particularly in the ATR prevention programme, and HIV patients adhere to ART, HIV transmission is prevented and reduced to negligible rates.

## Figures and Tables

**Table 1:** The spatio-temporal random effect linear predictor parameter and prior description.

Parameter	Parameter Definition	Prior Specification
α	This is the mean log overall LTFU risk over all regions.	The α parameter was assumed to follow a flat distribution, i.e., $\alpha \sim U\left(-\infty ,+\infty \right)$ to have a “sum to zero” constraint for the structured spatial parameter.
$X_{it}^{T}\beta$	This denotes the fixed effects regression coefficients $\beta =\left(\beta_{1},\beta_{2},\ldots ,\beta_{m}\right)$ associated with explanatory variables $\left(X_{i}^{T}=\left(X_{1},X_{2},\ldots ,X_{m}\right)\right)$.	The regression coefficients, $\beta =\left(\beta_{1},\beta_{2},\ldots ,\beta_{m}\right)$, were assumed to follow a non-informative Gaussian distribution with a mean $\left(\mu_{\beta }=0\right)$ and a wide variance, i.e., $\beta_{m}\sim iid\;N\left(\mu_{\beta },\sigma_{\beta }^{2}\right)$, with precision τβ=1σβ2.
$S_{i}$	The spatial random effects $S_{i}$ were partitioned into two components $\left(S_{i}=u_{i}+v_{i}\right)$ were $u_{i}$ defined as the structured spatial random effects that allow for smoothing amongst adjacent areas and $v_{i}$ defined as the unstructured spatial random effects to account for the extra-Poisson variability in the observed LTFU counts data [13].	The unstructured spatial random effects were assumed Gaussian priors, $v_{i}\overset{iid}{\sim }N\left(\mu_{v},\sigma_{v}^{2}\right)$, with precision τv=1σv2. The BYM model assumes spatial dependence between neighbouring areas; hence, the spatial polygons were assumed to follow a Gaussian distribution, i.e., $u_{i}|u_{-i}\sim N\left({\bar{u}}_{\delta_{i}},\frac{\sigma_{u}^{2}}{n_{\delta_{i}}}\right)$ where ${\bar{u}}_{\delta_{i}}$ is the mean parameter and $\sigma_{u}^{2}$ is the part of the variance parameter of the structured spatial component. The $n_{\delta_{i}}$ represents the number of neighbours and $\delta_{i}$ represents the sets of neighbours for the region i.
$\gamma_{t}$	This parameter defined the temporal random effects common to all regions.	The temporal random effects $\gamma_{t}$ were assumed first-order random walk priors $\gamma_{t}-\gamma_{t-1}\sim N\left(0,\tau_{\gamma }^{}\right)$t=2,…,Z and τγ=1σγ2.
$\psi_{it}$	This component defined the space-time interaction random effects that explain differences in the time trend of LTFU risk for different regions.	To investigate the space-time interaction, the $\psi_{it}$ was modelled as a Gaussian parameter with a precision matrix $\tau_{\psi }R_{\psi }$ where $\tau_{\psi }$ is an unknown scalar and $R_{\psi }$ is the correlation structure matrix defining the temporal and/or spatial dependence between the elements of ψ.

**Table 2:** The baseline characteristics of the study participants in each province.

Provinces	Sex (Females) N (%)	Age at ART Initiation Mean ± SD	Tuberculosis Infection (Positive) N (%)	WHO Clinical Stage (Stage III/IV) N (%)	Duration on ART Median(IQR)
Harare	9395 (65.77)	37.8 ± 10.6	363 (2.54)	7251 (50.76)	2.6 (1.2–5.8)
Bulawayo	5059 (63.0)	38.9 ± 10.9	297 (3.7)	3699 (46.06)	2.6 (1.1–5.6)
Manicaland	28,410 (66.07)	37.6 ± 11.4	465 (1.08)	25,872 (60.17)	3.7 (2.1–6.5)
Mashonaland Central	23,829 (66.09)	37.4 ± 11.5	225 (0.62)	23,696 (65.68)	3.1 (1.7–5.7)
Mashonaland East	36,584 (65.72)	36.8 ± 11.2	625 (1.12)	30,647 (54.99)	3.6 (2.1–5.7)
Mashonaland West	33,812 (64.72)	37.2 ± 11.1	583 (1.12)	31,218 (59.75)	2.8 (1.8–4.4)
Masvingo	39,614 (66.47)	37.9 ± 11.6	669 (1.12)	28,621 (48.02)	3.3 (1.9–5.7)
Matabeleland North	22,677 (63.73)	37.2 ± 11.8	436 (1.23)	16,463 (46.27)	3.6 (2.1–5.9)
Matabeleland South	25,977 (66.14)	36.7 ± 11.8	562 (1.43)	20,911 (53.24)	3.3 (1.9–5.3)
Midlands	30,487 (64.95)	37.6 ± 11.4	591 (1.26)	23,439 (49.94)	3.7 (2.1–6.2)

The categorical variables were summarised only for one category: sex (male/female); tuberculosis (negative/positive) and WHO clinical stage (I/II vs III/IV).

**Table 3:** Multiple variable Bayesian spatio-temporal Poisson regression models estimate the Loss to follow-up risk at the province level.

Variables	Province-Level Spatial Unit				
	Non-Spatio-Temporal Model RR (95%CI)	Spatio-Temporal Model RR (95%CI)	Type I Interaction Model RR (95%CI)	Type II Interaction Model RR (95%CI)	Type III Interaction Model RR (95%CI)
Sex					
Male	Reference	Reference	Reference	Reference	Reference
Female	0.914 (0.91–0.92)	0.975 (0.97–0.98)	0.915 (0.81–1.04)	0.976 (0.94–1.01)	1.011 (1.97–1.05)
Age at ART initiation					
(mean age in years)	0.601 (0.68–0.71)	0.818 (0.79–0.84)	1.092 (0.79–1.51)	1.004 (1.00–1.01)	1.014 (0.97–1.06)
Tuberculosis co-infection					
No	Reference	Reference	Reference	Reference	Reference
Yes	0.747 (0.74–0.76)	0.616 (0.61–0.63)	0.700 (0.59–0.83)	0.895 (0.77–1.05)	0.701 (0.61–0.81)
WHO staging					
I/II	Reference	Reference	Reference	Reference	Reference
III/IV	1.025 (1.02–1.03)	1.014 (1.01–1.02)	0.967 (0.93–1.00)	0.993 (0.97–1.01)	0.981 (0.96–1.00)
Duration on ART					
(average time since ART initiation	0.601 (0.59–0.61)	0.742 (0.71–0.77)	0.618 (0.44–0.85)	0.651 (0.59–0.71)	0.736 (0.56–0.97)
Information criterion					
DIC	9367.17	3079.93	787.33	784.08	789.36
WAIC	21,338.73	6647.31	768.86	766.81	772.89
pD	3677.72	1222.38	78.08	41.95	44.48
Marginal log-likelihood	−8520.78	−2699.27	−635.33	−600.38	−628.81

DIC = Deviance Information Criterion, WAIC = Watanabe-Akaike information criterion; pD = number of parameters; All interaction models had spatio-temporal random effects; RR = relative risk; Significance was set at 5%.

## Data Availability

The data used for this study can be found from a third party through an application process to the Zimbabwe Ministry of Health and Child Care through the HIV/AIDS Unit which oversees the data collection and compilation process for the ART program; therefore, the data is not publicly available.

## Supplementary Materials

The following are available online at https://www.mdpi.com/article/10.3390/ijerph191711013/s1, Section S1: Posterior model derivation, Section S2: R code, Figure S1: Temporal maps showing the observed LTFU rates at the district level in Zimbabwe, 2009–2016, Figure S2: Temporal maps showing the predicted LTFU rates at the district level in Zimbabwe, 2009–2016.
