# Supplementary material for: Loss to Follow-Up Risk among HIV Patients on ART in Zimbabwe, 2009–2016: Hierarchical Bayesian Spatio-Temporal Modeling
Source: Int J Environ Res Public Health. 2022 Sep 2;19(17):11013. doi: 10.3390/ijerph191711013 (PMC9518110; doi:10.3390/ijerph191711013)
Supplement: Supplementary file 1 [file ijerph-19-11013-s001.zip › ijerph-1853964-supplementary.pdf]

Section S1: Posterior model derivation

Likelihood specification

Let the LTFU counts follow a Poisson distribution with the following probability density function:

$$P(Y_{it}) = \frac{(\lambda_{it}E_{it})^{Y_{it}}}{Y_{it}!}\exp(-\lambda_{it}E_{it}) \text{ ; } i = 1,2,...,n \text{ and } t = 1,2,...,Z$$
$$\therefore Y_{it} \sim \text{Poisson}(\lambda_{it}E_{it}) \tag{A.1}$$

where Y_{it} defines the observed LTFU counts at the region i at the time t. The model assumes that the mean of the observed LTFU counts (μ_{it}) is a product of the expected count (E_{it}) and the relative risk (λ_{it}), i.e $\mu_{it} = \lambda_{it}E_{it}$.

The likelihood of the data is defined as:

$$\begin{aligned} P(\mathbf{Y}_{it} \mid \boldsymbol{\lambda}_{it}) &= P(Y_{1t}, Y_{2t}, ..., Y_{nt} \mid \lambda_{1t}, \lambda_{2t}, ..., \lambda_{nt}) \\ &= \prod_{t=1}^{Z}\prod_{i=1}^{n}\left[\frac{(\lambda_{it}E_{it})^{Y_{it}}}{Y_{it}!}\exp(-\lambda_{it}E_{it})\right] \\ &\propto \sum_{t=1}^{Z}\sum_{i=1}^{n}(\lambda_{it}E_{it})^{Y_{it}}\, nZ\left[\exp(-\lambda_{it}E_{it})\right] \end{aligned} \tag{A.2}$$

Taking the log-likelihood of equation 3, differentiation with respect to λ_{it} gives

$$\begin{aligned} \log(\lambda_{it}E_{it}) &= \log\left[(\lambda_{it}E_{it})^{\sum_{t=1}^{Z}\sum_{i=1}^{n}Y_{it}}\right] - (\lambda_{it}E_{it})nZ \\ &= \sum_{t=1}^{Z}\sum_{i=1}^{n}\left[Y_{it}(\log\lambda_{it} + \log E_{it}) - \lambda_{it}E_{it}\right] \end{aligned}$$

$$\begin{aligned} \frac{\partial \log(\lambda_{it}E_{it})}{\partial \lambda_{it}} &= \sum_{t=1}^{Z}\sum_{i=1}^{n}\left(\frac{Y_{it}}{\lambda_{it}} - E_{it}\right) = \sum_{t=1}^{Z}\sum_{i=1}^{n}\left(\frac{Y_{it}}{\lambda_{it}}\right) - \sum_{t=1}^{Z}\sum_{i=1}^{n}(E_{it}) \\ &= \frac{nZY_{it}}{\lambda_{it}} - nZE_{it} \\ &= Zn\left(\frac{Y_{it}}{\lambda_{it}} - E_{it}\right) \end{aligned} \tag{A.3}$$

Setting the derivative to zero gives a maximum likelihood estimate of the rate

$$Zn\left(\frac{Y_{it}}{\lambda_{it}}-E_{it}\right)=0$$
$$\frac{Y_{it}}{\lambda_{it}}-E_{it}=0 \tag{A.4}$$
$$\hat{\lambda}_{it}=\frac{Y_{it}}{E_{it}}$$

This likelihood estimate defines the crude yearly Standardised Incidence Ratios (SIRs) of the LTFU counts region i at the time t. The use of SIRs is the standard maximum likelihood approach which can be used to describe spatial heterogeneity of the outcome; however, in instances where very extreme values occur in regions with small populations due to the small sample sizes are involved; this may result in the overestimation or underestimation of the associated outcome risk. Moreover, if the likelihood is complex and the number of parameters is large, this approach may become difficult to implement. To overcome this limitation, Bayesian inferential models are preferred to obtain the outcome risk estimates accounting for confounding factors; adjusting for the neighbouring areas' random effects and parameter estimates can be smoothed or shrunk to improve the precision [20].

The linear predictor model specification

The linear predictor model describes the underlying structure of the relative risk in relation to the fixed and random effects. The linear predictor model included the Spatio-temporal random effects as defined by the convolutional conditional autoregressive (CAR) model proposed by Besag-York-Mollie`(BYM) [17] with separable spatial structured and spatial unstructured random effects; and temporal components. The model is specified as follows:

$$\log(\lambda_{it})=\log(E_{it})+\alpha+\boldsymbol{X}_i^T\boldsymbol{\beta}+u_i+v_i+\gamma_t+\psi_{it} \tag{A.5}$$

where α is the mean log overall LTFU risk over all regions, $\boldsymbol{X}_{it}^T\boldsymbol{\beta}$ denotes the fixed effects regression coefficients $\boldsymbol{\beta}=(\beta_1,\beta_2,...,\beta_m)$ associated with explanatory variables $\left(\boldsymbol{X}_i^T=X_1,X_2,...,X_m\right)$. The spatial random effects $\boldsymbol{S}_i$ are partitioned into two components $\left(\boldsymbol{S}_i=u_i+v_i\right)$ were u_i is structured spatial random effects that allow for smoothing amongst adjacent areas and v_i is unstructured spatial random effects. These spatial random effects accounted for the extra-Poisson variability in the observed LTFU counts data[20]. The component γ_t defines the overall temporal random effects common to all regions. The ψ_{it} defines the space-time interaction that explains differences in the time trend of LTFU risk for different regions.

Prior specification

All unknown parameters and hyper-priors were assigned some prior information. The α parameter was assumed to follow a Uniform distribution, i.e $\alpha\sim U\left(-\infty,+\infty\right)$ to have a “sum

to zero" constraint for the structured spatial parameter. The regression coefficients, $\boldsymbol{\beta}=(\beta_1,\beta_2,...,\beta_m)$, were assumed to follow a non-informative Gaussian distribution with a mean $(\mu_\beta=0)$ and a wide variance, i.e $\beta_m \sim iid\ N(\mu_\beta,\sigma_\beta^2)$, with a precision of $\tau_\beta = 1/\sigma_\beta^2$.

$$P(\beta_m)=\frac{1}{\sqrt{2\pi\sigma_\beta^2}}\exp\left[-\frac{1}{2}\left(\frac{\beta_m-\mu_\beta}{\sigma_\beta}\right)^2\right]$$

$$P(\beta_m \mid \tau_\beta)=\frac{1}{\sqrt{2\pi/\tau_\beta}}\exp\left[-\frac{1}{2}\left(\frac{\beta_m-\mu_\beta}{1/\sqrt{\tau_\beta}}\right)^2\right] \text{ ; for } \mu_\beta=0 \ \& \ \tau_\beta = 1/\sigma_\beta^2 \tag{A.6}$$

The unstructured spatial random effects were assumed Gaussian priors, $v_i \overset{iid}{\sim} N(\mu_v,\sigma_v^2)$ where σ_v^2 is the variance component specified as a precision $\tau_v = 1/\sigma_v^2$.

$$P(v_i \mid \sigma_v^2)=\frac{1}{\sqrt{2\pi\sigma_v^2}}\exp\left[-\frac{1}{2}\left(\frac{v_i-\mu_v}{\sigma_v}\right)^2\right]$$

$$P(v_i \mid \tau_v)=\frac{1}{\sqrt{2\pi/\tau_v}}\exp\left[-\frac{1}{2}\left(\frac{v_i-\mu_v}{1/\sqrt{\tau_v}}\right)^2\right] \text{ ; for } \mu_v=0 \ \& \ \tau_v = 1/\sigma_v^2 \tag{A.7}$$

The BYM model assumes spatial dependence between neighbouring; hence, the spatial polygons were assumed to follow a Gaussian distribution, i.e,

$$u_i \mid u_{-i} \sim N\left(\bar{u}_{\delta_i},\frac{\sigma_u^2}{n_{\delta_i}}\right)$$

$$P(u_i \mid \sigma_u^2)=\frac{1}{\sqrt{2\pi\left(\sigma_u^2/n_{\delta_i}\right)}}\exp\left[-\frac{1}{2}\left(\frac{\bar{u}_{\delta_i}-\mu_u}{\sigma_u/\sqrt{n_{\delta_i}}}\right)^2\right]$$

$$P(u_i \mid \tau_u) = \frac{1}{\sqrt{2\pi\left(\frac{1}{\tau_u n_{\delta_i}}\right)}} \exp\left[-\frac{1}{2}\left(\frac{\bar{u}_{\delta_i} - \mu_u}{\frac{1}{\sqrt{\tau_u n_{\delta_i}}}}\right)^2\right] \quad \text{for } \tau_u = \frac{1}{\sigma_v^2} \qquad \text{(A.8)}$$

where $\bar{u}_{\delta_i}$ is the mean parameter and σ_u^2 is the part of the variance parameter of the structured spatial component. The n_{δ_i} represents the number of neighbours and δ_i represents the sets of neighbours for the region i.

The temporal random effects γ_t were assumed first-order random walk priors $\gamma_t - \gamma_{t-1} \sim N(0, \tau_\gamma)$ $t = 2,...,Z$ with a precision of .

$$P(\gamma_i \mid \tau_\gamma) = \frac{1}{\sqrt{2\pi\left(\frac{1}{\tau_\gamma}\right)}} \exp\left[-\frac{1}{2}\left(\frac{\mu}{\frac{1}{\sqrt{\tau_\gamma}}}\right)^2\right] \quad \text{for } \tau_\gamma = \frac{1}{\sigma_\gamma^2} \text{ (A.9)}$$

To investigate the space-time interaction, the ψ_{it} was modelled as a Gaussian parameter with a precision matrix $\tau_\psi \mathbf{R}_\psi$ where τ_ψ is an unknown scalar and $\mathbf{R}_\psi$ is the correlation structure matrix defining the temporal and/or spatial dependence between the elements of ψ.

The posterior estimates

The posterior estimates were obtained through the marginal distribution of each parameter to be estimated. In other words, the posterior estimates are obtained by multiplying the likelihood and the prior distributions.

$$P(\Omega \equiv \alpha, \boldsymbol{\beta}, \boldsymbol{\upsilon}_i, v_t, \gamma_{it} \mid Y_{it}) \propto L(\boldsymbol{Y}_{it} \mid \gamma_{it}) \times P(\beta_m) \times P(u_i \mid \tau_u) \times P(v_i \mid \tau_v) \times P(\gamma_{it} \mid \tau_\gamma)$$
$$= \sum_{t=1}^{Z} \sum_{i=1}^{n} (\lambda_{it} E_{it})^{Y_{it}} nZ\left[\exp(-\lambda_{it} E_{it})\right] \qquad \text{(A.10)}$$
$$\times \frac{1}{\sqrt{2\pi / \tau_\beta}} \exp\left[-\frac{1}{2}\left(\frac{\beta_m - \mu_\beta}{\frac{1}{\sqrt{\tau_\beta}}}\right)^2\right]$$

$$
\times\frac{1}{\sqrt{2\pi\left(\frac{1}{\tau_u n_{\delta_i}}\right)}}\exp\left[-\frac{1}{2}\left(\frac{\bar{u}_{\delta_i}-\mu_u}{1/\sqrt{\tau_u n_{\delta_i}}}\right)^2\right]
$$

$$
\times\frac{1}{\sqrt{2\pi/\tau_v}}\exp\left[-\frac{1}{2}\left(\frac{v_i-\mu_v}{1/\sqrt{\tau_v}}\right)^2\right] \tag{A.11}
$$

$$
\times\frac{1}{\sqrt{2\pi\left(\frac{1}{\tau_\gamma}\right)}}\exp\left[-\frac{1}{2}\left(\frac{\mu}{1/\sqrt{\tau_\gamma}}\right)^2\right]
$$

Since this posterior function has no closed-form, we used the using R Integrated Nested Laplace Approximation (INLA) package, which is less computationally intensive compared to the Markov Chain Monte Carlo (MCMC) methods [6].

Section S2: R code

```
    library(spdep)
    library(maptools)
    library(foreign)
    library(sp)
    library(rgdal)
    library(lattice)
    library(R2BayesX)
    library(R2WinBUGS)
    library(shapefiles)
    library(BayesX)
##########################################################################
####SPatio-temporal model

    model1<- inla(LTFU~TB_status+ WHO_stage+ Sex+ Time_since_ART_initiation+
    Age_at_ART_initiation+
            f(Year, model = "rwl",hyper = prec_period)+
            f(Time, model = "iid")+
            f(ID1, model = "besag", graph =ZimADM1adj.mat,hyper = prec_space),
            data = DataProv, family ="poisson",E=Expected,
           list(return.marginals.random=TRUE,return.marginals.predictor=TRUE),
           control.compute=list(dic=TRUE,cpo=TRUE,waic=TRUE))

    summary(model1)
```

```
#################################################################################
####Spatial and temporal are independent

DataProv$ProvYear<-DataProv$Province*DataProv$Year

model2<- inla(LTFU~TB_status+ WHO_stage+ Sex+ Time_since_ART_initiation+
Age_at_ART_initiation +
            f(Year, model = "rw1",hyper = prec_period)+
            f(Time, model = "iid")+
            f(ProvYear, model = "iid")+
            f(ID1, model = "besag", graph =ZimADM1adj.mat,hyper = prec_space),
          data = DataProv, family ="poisson",E=Expected,
          list(return.marginals.random=TRUE,return.marginals.predictor=TRUE),
          control.compute=list(dic=TRUE,cpo=TRUE,waic=TRUE))
summary(model2)

#################################################################################
##Interaction term is temporally correlated with each spatial unit
#Time trends in different areas are independent

Province.int <- DataProv$Province
Year.int <- DataProv$Year2
model3<- inla(LTFU~TB_status+ WHO_stage+ Sex+ Time_since_ART_initiation+
Age_at_ART_initiation +
            f(Year, model = "rw1",hyper = prec_period)+
            f(Time, model = "iid")+
            f(Province.int,model="iid", group=Year.int,control.group=list(model="rw1"),hyper =
prec_interact)+
            f(ID1, model = "besag", graph =ZimADM1adj.mat,hyper = prec_space),
          data = DataProv, family ="poisson",E=Expected,
          list(return.marginals.random=TRUE,return.marginals.predictor=TRUE),
          control.compute=list(dic=TRUE,cpo=TRUE,waic=TRUE))
summary(model3)
###################################################################################
###Unstructured temporal effects with a spatial structured effect

model4<- inla(LTFU~TB_status+ WHO_stage+ Sex+ Time_since_ART_initiation+
Age_at_ART_initiation +
            f(Year, model = "rw1",hyper = prec_period)+
            f(Time, model = "iid")+
            f(Year.int,model="iid",group=Province.int,
              control.group=list(model="besag", graph=ZimADM1adj.mat),hyper = prec_uspace)+
            f(ID1, model = "besag", graph =ZimADM1adj.mat,hyper = prec_space),
          data = DataProv, family ="poisson",E=Expected,
          list(return.marginals.random=TRUE,return.marginals.predictor=TRUE),
          control.compute=list(dic=TRUE,cpo=TRUE,waic=TRUE))
summary(model4)
```

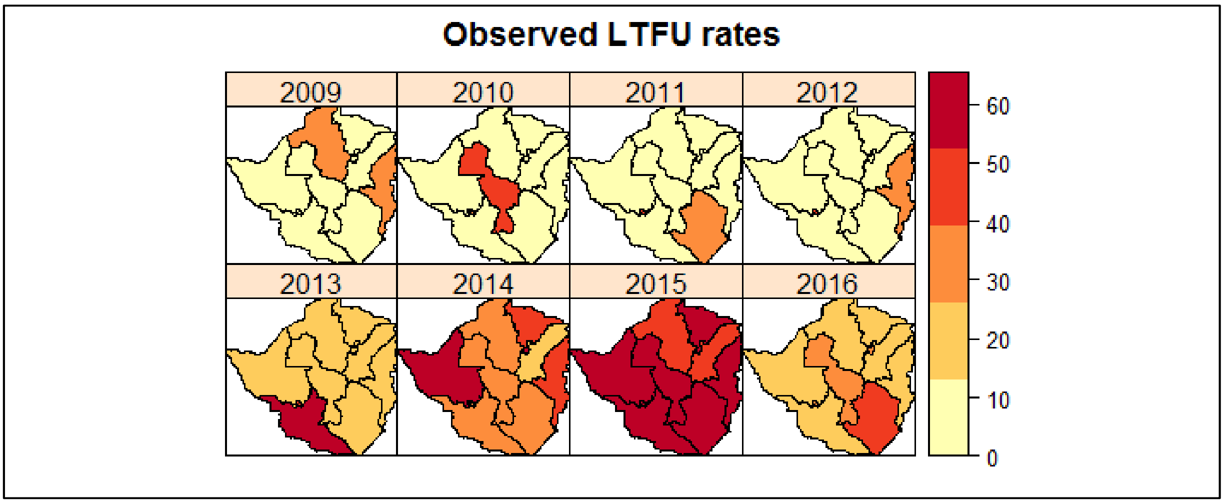

Figure S1: Temporal maps showing the observed LTFU rates at the district level in Zimbabwe, 2009-2016

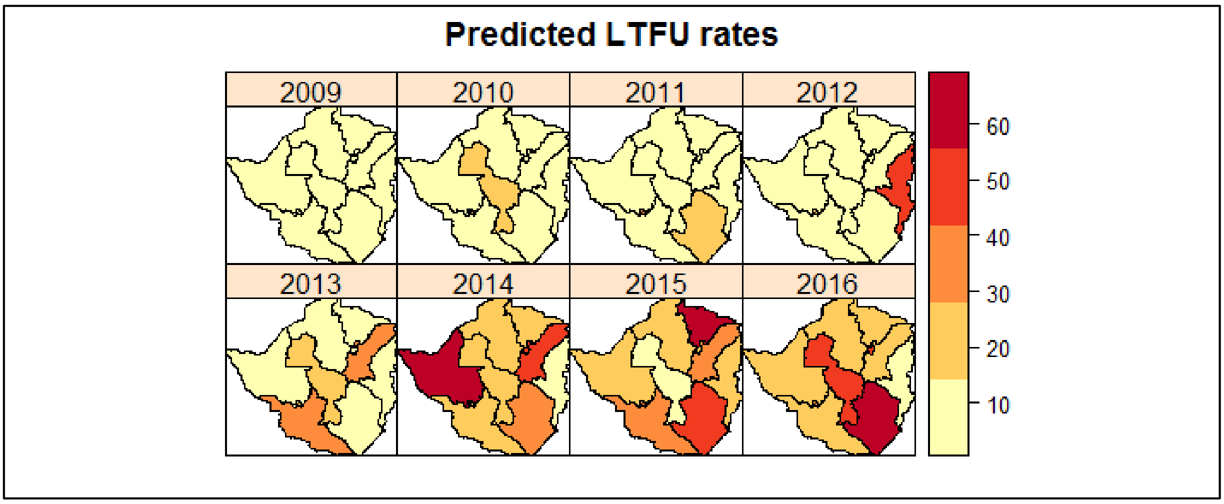

Figure S2: Temporal maps showing the predicted LTFU rates using the Poisson model at the district level in Zimbabwe, 2009-2016
